# Non-Contact Vibro-Acoustic Object Recognition Using Laser Doppler Vibrometry and Convolutional Neural Networks

**DOI:** 10.3390/s22239360

**Published:** 2022-12-01

**Authors:** Abdel Darwish, Benjamin Halkon, Sebastian Oberst

**Affiliations:** Centre for Audio, Acoustics and Vibration, University of Technology Sydney, Ultimo, NSW 2007, Australia

**Keywords:** vibratory response, vibro-acoustic classification, acoustic fingerprint, non-contact excitation, transfer learning, deep learning

## Abstract

Laser Doppler vibrometers (LDVs) have been widely adopted due to their large number of benefits in comparison to traditional contacting vibration transducers. Their high sensitivity, among other unique characteristics, has also led to their use as optical microphones, where the measurement of object vibration in the vicinity of a sound source can act as a microphone. Recent work enabling full correction of LDV measurement in the presence of sensor head vibration unlocks new potential applications, including integration within autonomous vehicles (AVs). In this paper, the common AV challenge of object classification is addressed by presenting and evaluating a novel, non-contact vibro-acoustic object recognition technique. This technique utilises a custom set-up involving a synchronised loudspeaker and scanning LDV to simultaneously remotely solicit and record responses to a periodic chirp excitation in various objects. The 864 recorded signals per object were pre-processed into spectrograms of various forms, which were used to train a ResNet-18 neural network via transfer learning to accurately recognise the objects based only on their vibro-acoustic characteristics. A five-fold cross-validation optimisation approach is described, through which the effects of data set size and pre-processing type on classification accuracy are assessed. A further assessment of the ability of the CNN to classify never-before-seen objects belonging to groups of similar objects on which it has been trained is then described. In both scenarios, the CNN was able to obtain excellent classification accuracy of over 99.7%. The work described here demonstrates the significant promise of such an approach as a viable non-contact object recognition technique suitable for various machine automation tasks, for example, defect detection in production lines or even loose rock identification in underground mines.

## 1. Introduction

Laser Doppler vibrometers (LDVs) have numerous advantages over traditional contacting transducers, such as having higher spatial resolution, frequency bandwidth and dynamic range [1]. Their optical, inherently non-contact nature enables remote, non-invasive target vibration measurements, leading to a range of specific benefits in various fields. For example, measurements on lightweight structures particularly benefit from zero mass loading, especially for thin-walled elastic structures [2]. Furthermore, standoff distances of up to hundreds of meters enable measurements from targets in otherwise inaccessible or particularly hazardous areas. Examples include operational wind turbine [3] and turbomachinery [4] dynamic assessment, and buried land mine detection [5]. In the former examples, the probe laser beam is directed toward and maintained on the point of interest on the target during operation, while in the latter, the scattering of ground surface waves around objects of interest are imaged and interrogated with scanning LDVs.

With computational power increasing seemingly unabated, the application of machine learning to the interpretation of structural vibration and acoustical measurement data has attracted significant interest [6,7,8]. Recent examples include the use of ground-penetrating radar data and a convolutional neural network (CNN) to detect the presence of a landmine [9], recurrence quantification analysis to detect objects buried in the seabed from raw sonar data [10] and machine learning to perform speaker diarisation from LDV measurements [11]. It is quite straightforward to imagine the extension of these various contemporary vibro-acoustic detection, localisation and classification techniques into solutions that can be deployed on or from autonomous vehicles, especially since this immediately extends their application to scenarios in hostile environments that are not suitable for human presence. However, with this comes the concomitant requirement to remove the effect of any vibration of the platform on which the sensor suite is mounted. In the case of LDVs, which are equally sensitive to instrument vibration as to the target vibration, recent advances have shown that by using the data obtained from correctly positioned measurement transducers on the LDV or steering optics, the target vibration can be fully recovered [12,13]. While the mobile deployment of LDVs on autonomous vehicles is yet to be realised, their successful integration would augment capability in many in-field applications that have already received interest, for example, buried landmine detection from mobile platforms [14], orbital seismology [15,16,17], terrestrial seismology [18] and structural health monitoring from drones [19], thereby, positioning LDVs as powerful yet underutilised tools for mobile deployment on autonomous systems.

In this context, this work presents a new addition to the ever-growing body of LDV applications: non-contact vibro-acoustic object recognition. Up until now, existing acoustic object recognition approaches described in the literature have tended to involve excitation techniques that are *contact* in nature, using either a simple actuator [20,21] or a multiple degrees-of-freedom robotic arm [22,23] to excite an audible response in the object that is measured by a microphone. These recorded sounds are then classified using a range of signal processing and machine learning techniques. Simple actuators have been applied to excite filled containers using a shake motion [20,21], whereas robotic arms have used various exploratory behaviours such as *lift, shake, drop, crush and push* motions in order to be compatible with a broader range of objects [22,23]. All these techniques have focused mainly on the identification of household objects and have obtained accuracies as high as 96%. However, the act of exciting an object via touch has some drawbacks. Firstly, this requires physical access to the object, meaning the robot must move towards the object, making the task slower and more complex. Secondly, the object must be excited with sufficient force to produce an audible response. Therefore, some fragile objects could be damaged during the excitation, for example, *dropping* an object made from glass would probably not be sensible. The new vibro-acoustic object recognition technique presented herein addresses these drawbacks by proposing an entirely non-contact technique based on an LDV.

This new *non-contact* vibro-acoustic object recognition technique substitutes the contacting actuator of the robot with an acoustic excitation generated by a loudspeaker. However, the response generated as a result of this acoustic excitation is orders of magnitude lower in amplitude than those previously excited via touch, generating little to no detectable sound. These low-amplitude acoustically excited vibrations have been previously shown to be detectable for remote acoustic measurements using an LDV due to their inherently high dynamic range and sensitivity [24,25]. As such, the traditional microphone employed in previous research is substituted with an LDV acting as an *optical* microphone, directly measuring the low-amplitude vibrational response of the object itself, rather than remotely measuring the sound the object generates as a result of the excitation. The recorded responses were processed with a range of spectrogram-based techniques, which were then used to train a CNN via transfer learning. A rigorous five-fold cross-validation was used to optimise the required training set size and pre-processing technique to show that accuracies in excess of 99.7% are obtainable when classifying 23 household objects. In order to extend the technique towards more practical applications, the ability of the technique to classify objects belonging to broader classes of objects was then evaluated to show the successful identification of never-before-seen instances of the class with similar near-perfect performance, for example, on variations of soda cans. This near-perfect classification performance is higher than the existing *contact* techniques, whilst simultaneously introducing valuable improved *non-contact* functionality. Therefore, this method is positioned as a viable object recognition technique for integration in autonomous systems and potentially even other machine automation tasks.

## 2. Data Collection and Preparation

### 2.1. Object Selection

**Figure 1:**
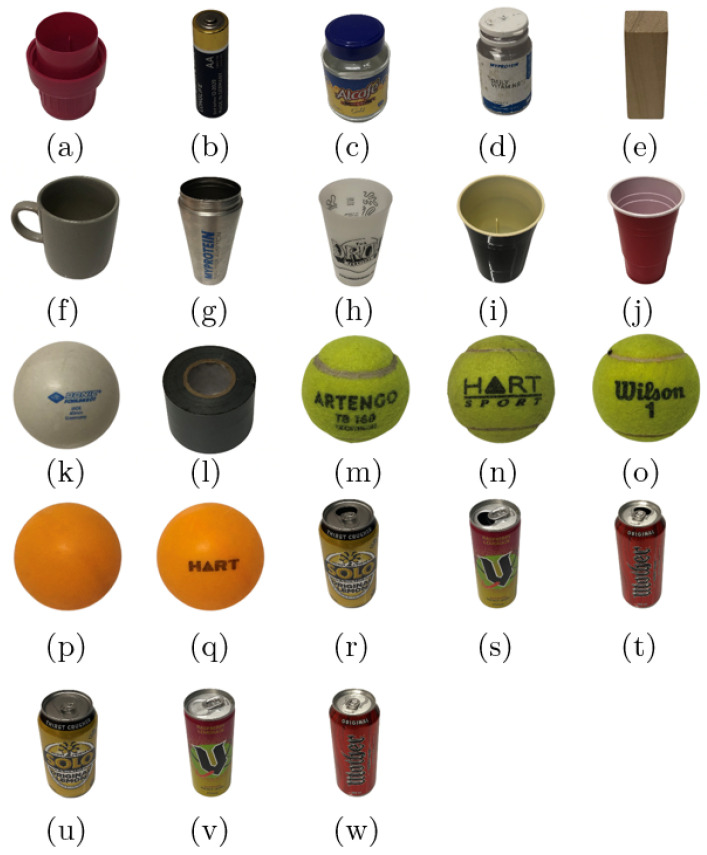
The 23 household objects characterised in this paper: (**a**) bottle cap, (**b**) AA battery, (**c**) empty jar, (**d**) empty container, (**e**) small wooden block, (**f**) porcelain mug, (**g**) metal cup, (**h**) plastic cup 1, (**i**) plastic cup 2, (**j**) plastic cup 3, (**k**) table tennis ball 1, (**l**) duct tape, (**m**) tennis ball 1, (**n**) tennis ball 2, (**o**) tennis ball 3, (**p**) table tennis ball 2, (**q**) table tennis ball 3, (**r**) empty 375 mL soda can, (**s**) empty 250 mL soda can, (**t**) empty 500 mL soda can, (**u**) sealed 375 mL soda can, (**v**) sealed 250 mL soda can, (**w**) sealed 500 mL soda can. The empty soda cans were drained of their liquid, while the sealed soda cans were full of soda.

This work was concerned with the vibro-acoustic characterisation of 23 household objects shown in Figure 1. The objects were selected to satisfy the following three main criteria. Firstly, objects were selected to enable this research to remain within the scope of previous object recognition works [20,21,22,23]. Thus, the objects used are comprised of various common materials including ceramic, glass, plastic, metal and wood. Secondly, triplets of similar objects were included to assess the sensitivity of the approach to similar objects and its ability to generalise across broader object classes. As such, there are four main subgroups composed of three similar objects: (i) table tennis balls, (ii) tennis balls and (iii) full or (iv) empty soda cans. For example, the system could either distinguish the three tennis balls from one another *or* it could identify that a never-before-seen tennis ball is indeed a tennis ball. Finally, since a rotary stage is used to automatically collect vibro-acoustic data for each object, the objects must all possess some degree of axial symmetry and be small and light enough to fit on and be manipulated by the rotary stage.

### 2.2. Automated Data Acquisition System

**Figure 2:**
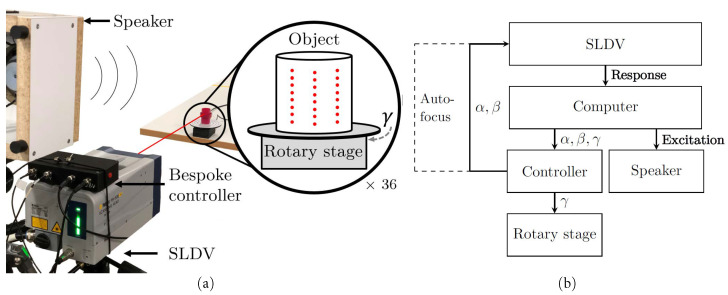
Experimental arrangement: (**a**) physical set-up with the sound source, measurement beam, and SLDV measurement grid highlighted, (**b**) block diagram where solid and dashed lines are wired and wireless connections, respectively, α and β are the scanning mirror angle command signals, and γ is the rotary stage angle command signal.

When applying deep learning to a new problem, it is difficult to know in advance how much data is required to achieve a certain performance outcome. Therefore, a large number of responses were collected for each object such that the optimal volume of training data could also be determined. This was tested by only using subsets of the full collected data to simulate the availability of only smaller data sets. A bespoke automated data collecting system, as shown in Figure 2, was developed to rapidly enable the collection of hundreds of measurements per object. Both the rotary stage and the controller were custom-built for this application. The rotary stage consisted of an Arduino Nano and a 28BYJ-48 stepper motor, both contained in a 3D-printed housing. A 3D-printed turntable attached to the motor shaft allowed the samples to be rotated about a vertical axis in front of the speaker and SLDV. Similarly, the controller consisted of an ESP-32 microcontroller, two 12-bit DACs and an op-amp circuit, all contained inside a custom 3D-printed housing. Two ±3 V analogue outputs—one per scanning mirror—and a single digital output to the rotary stage enabled control of the target rotation and SLDV beam orientation.

**Figure A1:**
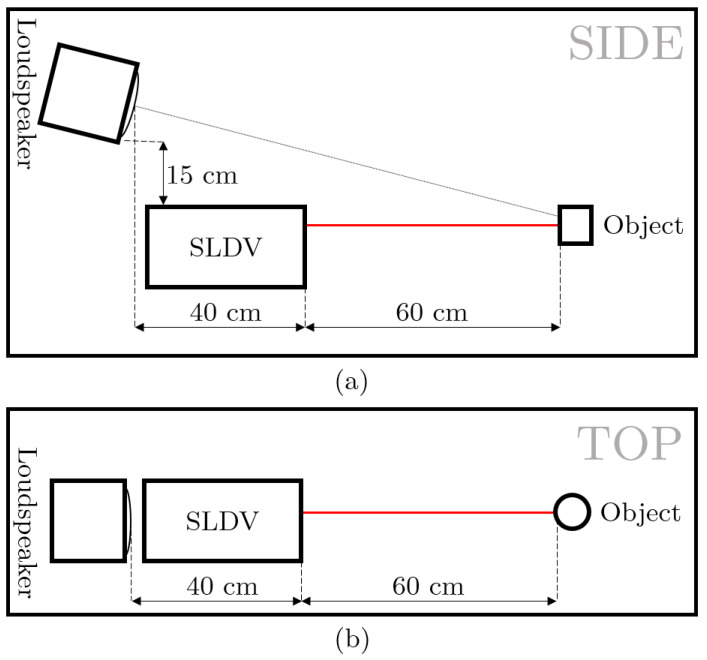
(**a**) side view and (**b**) top view schematic of the test apparatus with the relevant approximate dimensions annotated and the measurement beam highlighted in red.

Since LDVs measure the surface velocity of a target at a specific location in the direction of the laser beam, any change of scan location or beam angle will change the nature of the measurement and could adversely affect the subsequent object classification accuracy. While the object location is fixed in this work, eventual practical application of this technique to autonomous systems would need to be insensitive to the relative object–robot positioning. As such, care was taken to ensure that the training data was sufficiently diverse to be representative of a real application scenario by moving both SLDV steering mirrors and the rotary stage to collect a total of 864 responses at various locations and angles of incidences. A schematic of the setup with the relevant dimensions labelled can be seen in Figure A1. The responses were collected at a 0.6 m stand-off distance over an 8×3 grid for every position of the rotary stage. The height and width of this grid on each object was chosen to be 80% the total height or width of the object. Once measurements at each of the 24 grid points were acquired, the rotary stage was adjusted by 10 degrees and another 8×3 scan of measurements was collected. This procedure was repeated until the 8×3 scans were collected in 10-degree increments around the entirety of the object, giving 864 scans and taking approximately 20 min in total per object.

The entire operation was orchestrated by a Python script running on a laptop (HP Elite Book HSN-I13C-4; Intel Core i7-8650U @ 2.1 GHz, 16 G RAM). The laptop sent angle commands to the controller via a USB serial link; the controller then applied the corresponding voltages to the External Scanner Control (EXT) to adjust the mirror positions, and relayed the required object orientation angle to the rotary stage; the EXT is an optional extra on some Polytec scanning-laser Doppler vibrometers. The acoustic excitation imparted to the target was a 1 s long, 1 Hz to 20 kHz linear chirp played during each scan using a loudspeaker (Jaycar AS3007; Sydney, Australia; 125 mm; 5 Ω) via an amplifier module (Kemo Electronics M032N; Geestland, Germany; 12 W). This frequency range was chosen to be as broad as possible while still using a standard loudspeaker. The maximum A-weighted sound pressure level recorded at the target location was 67 dB re $20\times 10^{-6}$ Pa using a sound level meter (Digitech QM1592; Frankfurt, Germany). The resulting vibratory responses were recorded using the SLDV (Polytec PSV-500 Xtra Scanning Laser Vibrometer; Waldbronn, Germany; sensitivity: 306.2 mms^−1^V^−1^, sensitive range: 20–1250 kHz, velocity resolution: 0.12 μm/s). Both the excitation and response signals were played and recorded through the headphone and microphone jacks on the laptop for convenience, using a sampling frequency of 44.1 kHz.

### 2.3. Management of Measurement Challenges

Successful macro-scale LDV measurements generally rely on the target surface being rough on the scale of the optical wavelength. As a result, scattering of the inbound laser beam occurs, allowing sufficient light to be collected in direct backscatter in the instrument optics. Importantly, this allows measurements to be made even when the laser beam is not normal to the target surface. However, surface roughness also results in a de-phasing of the scattered monochromatic, coherent light, which, in turn, leads to the formation of a speckle pattern [26]. For a moving target or laser beam, the speckle pattern will change in sympathy, which can present a number of measurement challenges [1].

While state-of-art LDV systems include increasingly sophisticated attempts to overcome laser speckle challenges, these may not always be 100% effective. Furthermore, since externally generated signals controlled the scanning mirrors in this campaign, such features were not available as part of the acquisition. Another increasingly important feature of commercially available scanning LDVs is laser beam auto-focusing; this also contributes to optimising the chances of successful, automated measurements at a range of points across the surface of a structure. To maximise the collection of useful, high-quality measured signals in this campaign, a custom solution was developed to handle these two aspects.

**Figure 3:**
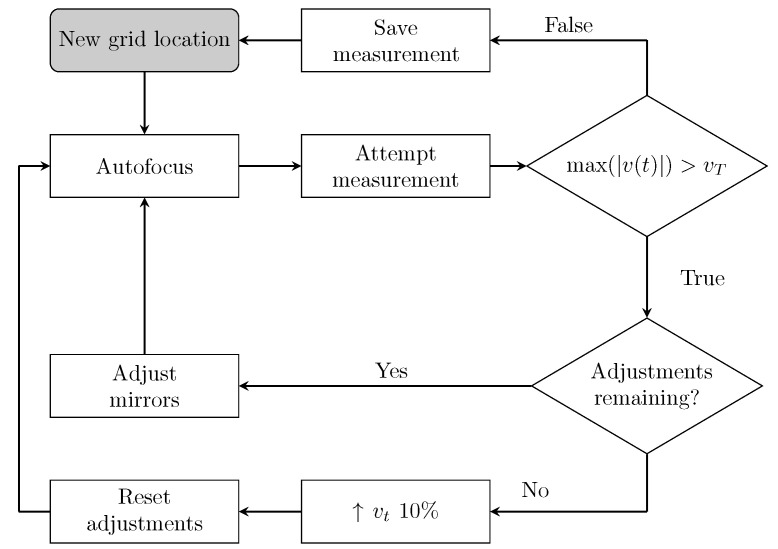
The measurement procedure where *t* is time, v(t) is the LDV measurement and $v_{T}$ is the threshold velocity, initially set to twice the root mean square of the first object measurement.

Figure 3 describes the measurement procedure from the moment after the laser beam has been directed to a new grid location on the target. First, the controller emulates a Bluetooth keyboard connected to the integrated PSV-500 Xtra Data Management System and issues a Ctrl + E keystroke to have the scanning head complete an auto-focus operation. With the laser beam focused at the point of interest, the speaker signal is output via the headphone jack and a recording is made using the LDV via the microphone jack. The level of this time recording, v(t), is interrogated and, should it exceed a threshold, $v_{T}$, it is rejected on the basis that a high-amplitude-inducing laser speckle noise “drop-out” might have occurred; otherwise, the measurement is saved. This threshold value is initially set to twice the root mean square of the first measurement of an object. In the case of a rejection, the laser beam position is fine-tuned by a 5 mrad scan angle adjustment sequentially in ±x or ±y and the process is repeated from the autofocus step until a satisfactory measurement is obtained. If the original location and the four adjusted locations fail to obtain a suitable measurement, it can be reasonably concluded that the threshold is too low and the system is incorrectly identifying measurements as containing speckle drop-outs. As such, the threshold value is increased by 10% to prevent the system from becoming stuck in an infinite loop and the processes is repeated. This revised threshold value is used for the entire object and will remain increased for all subsequent measurements, allowing the system to autonomously determine an appropriate value during the scans for each object. Finally, once a satisfactory measurement is obtained, the laser beam is directed to the next position on the grid or the target is rotated to the next rotary stage angle, as shown in Figure 2a.

### 2.4. Data Pre-Processing

**Figure 4:**
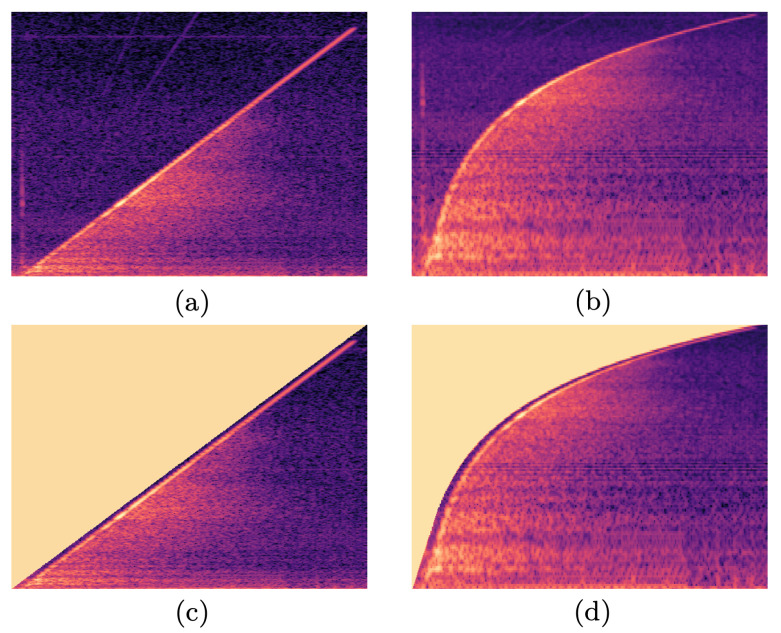
A response of object (j) shown in the four data types used to train the various CNNs; (**a**) spectrogram, (**b**) mel-spectrogram, (**c**) cropped spectrogram and (**d**) cropped mel-spectrogram. Axes are not presented to the CNN; similarly, the colours represent normalised amplitude.

**Figure A2:**
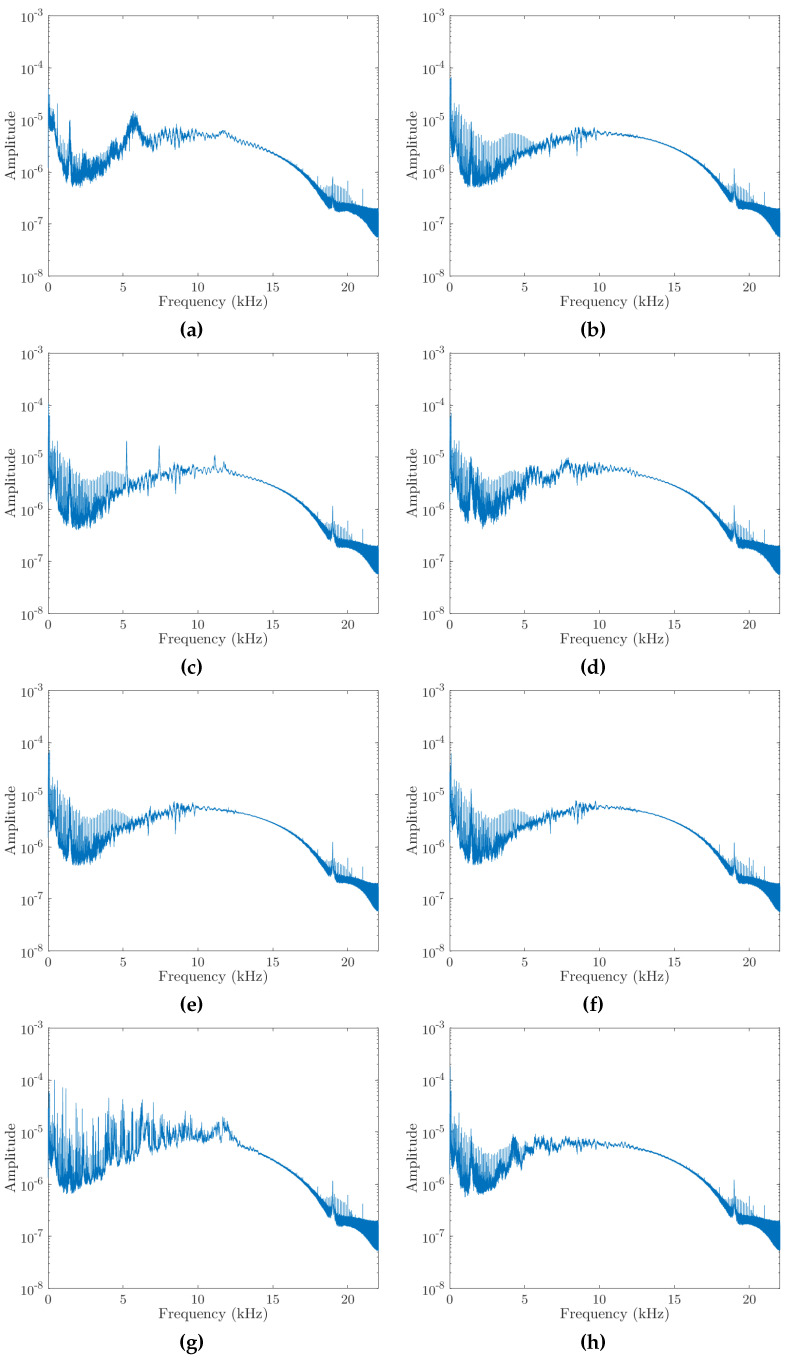

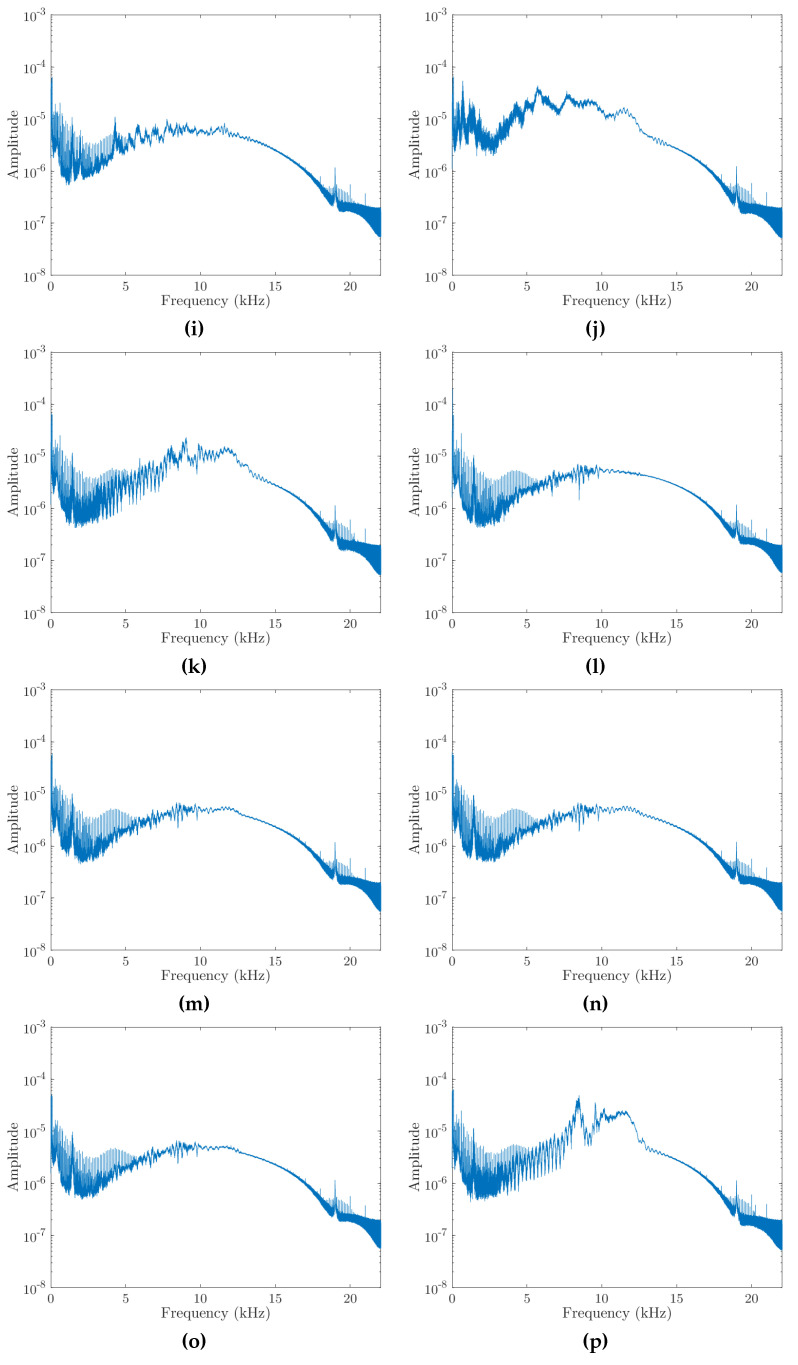

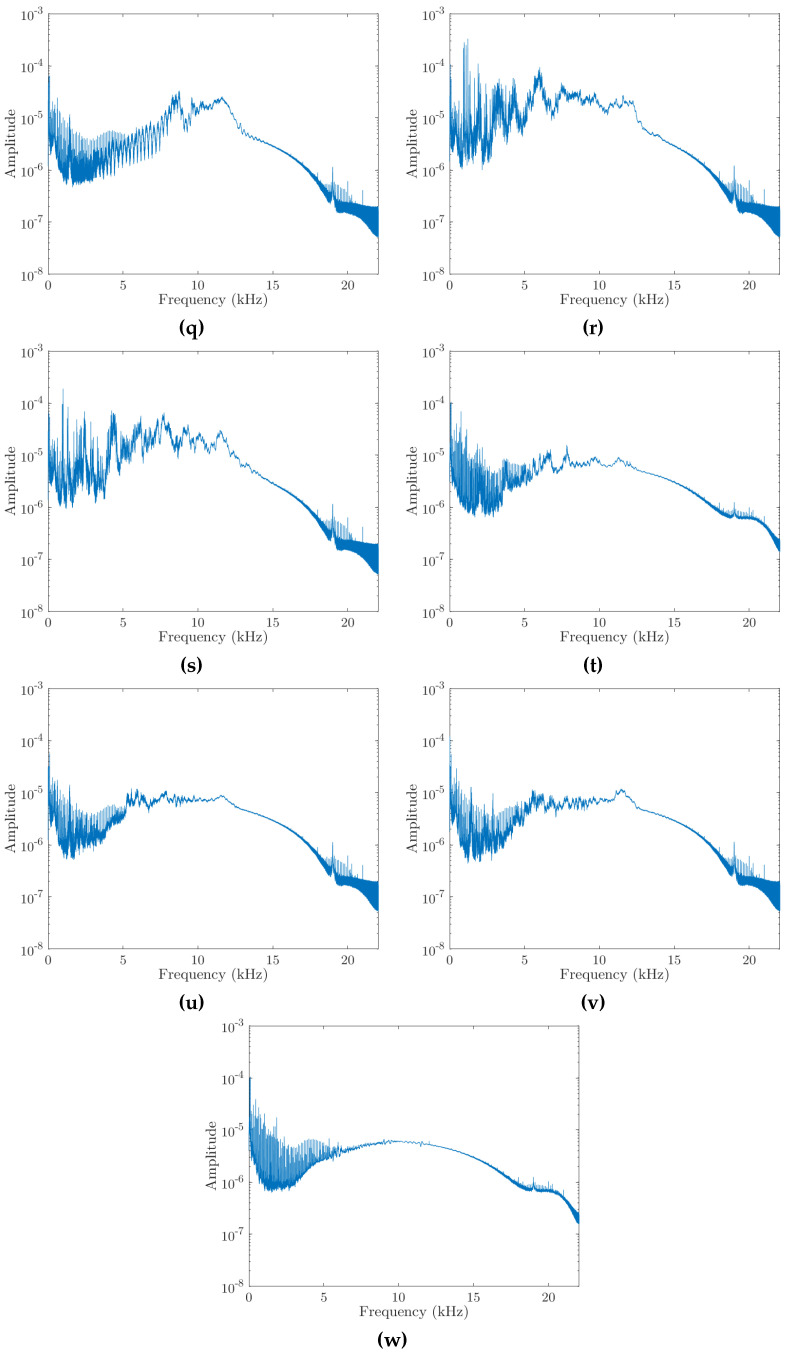
Mean spectra of all 864 Hann windowed responses for each object as measured through the laptop microphone port; as such, the amplitude has no units. Subfigures (**a**–**w**) corresponding to objects (**a**–**w**), respectively.

While the 864 responses per object were stored in the form of audio files (*.wav), these were all processed into images using the laptop for classification purposes to allow a broad range of available image processing NNs to be utilised. The raw measurements are publicly available via a link at the end of this paper, and the mean spectra for each object can be seen in Figure A2. Since pre-processing can have an effect on classification accuracy, this work will compare four similar but alternative pre-processing techniques. For each measurement, the first two options are spectrogram, showing the frequency content plotted against time, and mel-spectrogram, showing the frequency content on a non-linear scale (the mel scale). The mel-spectrogram uses a non-linear frequency scale based on human auditory perception and is a common pre-processing technique for audio classification-based tasks [27,28]. By taking the mean spectra of each object, shown in Figure A2, it can be noted that most of the object resonances occur in the lower frequencies. As such, mel-spectrograms are particularly well-suited for this application, since they devote more image area to these lower frequencies. With a focus on reducing the impact of measurement noise on classification accuracy, both spectrograms and mel-spectrograms are modified such that any spurious signal content above the excited frequency is removed; this will be referred to as “cropping” throughout this paper. This results in a total of four data types: spectrograms, mel-spectrograms, cropped spectrograms and cropped mel-spectrograms, as can be seen in Figure 4. Each of these will be used to train a CNN so that the efficacy of these data pre-processing techniques can be compared.

The pre-processing was implemented in Python using the Librosa library [29]. Each response was first used to generate a normalised spectrogram with a window size of 1024 samples, or 23.22 ms, which gives a spectral resolution of 0.043 Hz. The windows had an overlap of 6.25% and a maximum frequency bin of 22.05 kHz. The spectrograms were then “cropped” between opposing corners, shown in Figure 4c, removing any spectral information above the excitation frequency by setting these areas of the spectrogram to zero amplitude. Finally, the mel-spectrograms and the cropped mel-spectrograms were generated using their spectrogram counterparts with 150 bin frequencies. Convolutional neural networks (CNNs) were then trained and assessed on each of these four data types, which were in the form of 496 × 369-pixel images (*.png) as exported by Librosa.

## 3. Convolutional Neural Network Regularisation and Training

### 3.1. Summary of Fundamental Neural Network Concepts

**Figure 5:**
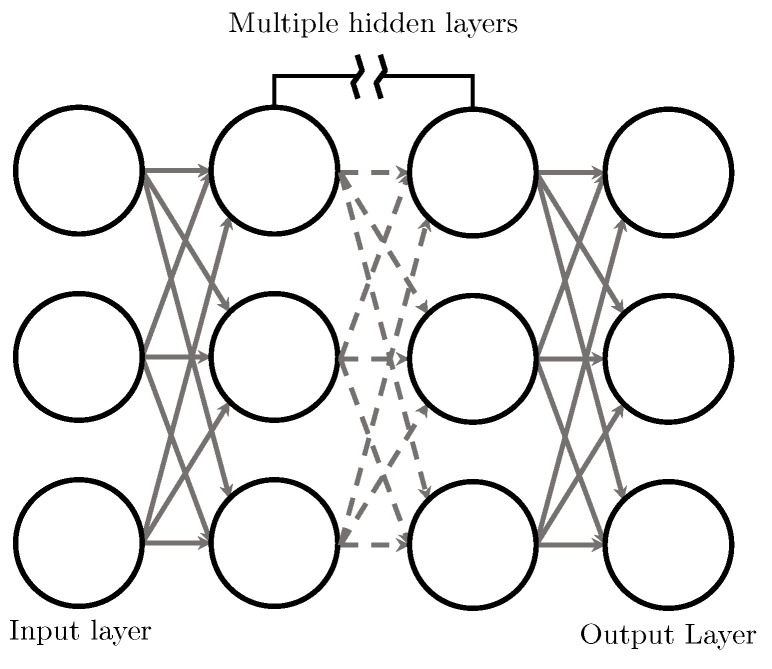
A simple schematic of an NN, with circles representing nodes and arrows representing how information is passed between them. The number of nodes in each layer is not necessarily equal.

CNNs are a type of neural network (NN) which specialise in processing data with a grid-like topology, and have therefore excelled at image recognition tasks [30]. Time domain audio data can be readily represented as images using a range of spectrogram-based techniques which maintain this three-dimensional grid-like topology; therefore, CNNs have been commonly used for audio classification tasks [28]. The name and fundamental structure of an NN is inspired by that of a biological brain, mimicking the way that neurons in a brain send signals to one another [31]. A simple schematic of a basic NN can be seen in Figure 5. The input nodes receive information, such as the brightness of a pixel (for a monochromatic image; otherwise, it is the brightness of a channel within a pixel), and pass this information on via a connection to a node in the next layer. Each node in the hidden layers uses the assigned weights of each connection and an internal bias to calculate an output value (activation functions are not mentioned here for simplicity) [32]. This process continues until the output layer, where the result can be acquired; for example, the classification of the image.

It is the weights and biases that determine the behaviour of an NN; they are selected through a process known as *training*. In the case of the supervised learning used herein, training is performed using a labelled data set, known as a *training set*. The training set is used to tune the weights and biases with the intention of matching the NN outputs to the correct labels. How exactly the weights and biases are tuned is regulated by various parameters, known as hyperparameters [32]. The correct selection of the hyperparameters for optimum performance is a vital part of training any NN. One such hyperparameter is how many epochs an NN is trained for, an epoch being a single iteration of the training data by the NN, since training involves making repeated iterations over the training data to arrive at the final weights and biases. Since the NN has only been trained on the training data, it may be successful within the training data set, but unsuccessful on an unseen data set; this is known as *overfitting* and can occur if an NN is trained for too many epochs.

### 3.2. Regularisation to Prevent Overfitting

While there are a number of techniques available to prevent overfitting, known as *regularisation*, this work uses early stopping [33]. Early stopping uses a second data set, known as the *validation set*. The NN is not trained on this data set, but it does make predictions on it. These predictions are used to determine if the NN has made meaningful generalisations that apply to the validation set, or if the NN is only able to make accurate predictions on data within the training set. Practically, this means selecting a number of epochs that minimises *both* training loss and validation loss, where loss is a number which captures the accuracy of the NN when making predictions on either data set.

Typically, a data set is either split into 80% training set, and 20% validation set, or, when particular rigour is required, split into 70% training set, 20% validation set and 10% *test set* [32]. The test set is set aside in the early stages and is not used during the training procedure. Therefore, this data set is the most representative when estimating the real-world performance of the trained NN, as the user may select hyperparameters that could similarly lead to overfitting on the validation set.

### 3.3. Training Methodology for Response Classification

Rather than training an NN from scratch, a transfer learning (TL) approach was applied. TL uses an NN that has already been trained for a different but related task and retrains it for the new task, thus “re-purposing” the NN. Since only the weights and biases closer to the output are tuned, the network retains some knowledge learned from the initial data set. TL has three main general advantages over training an NN from scratch [34]. Firstly, the initial accuracy, after one epoch of training, will be higher. Secondly, the rate of improvement with increasing epochs will be steeper. Finally, once the performance plateaus with increased epochs, it will still remain higher than traditional techniques. These advantages are due to the NN applying general knowledge learned from the pre-training data set to the new data set, which increases the contextual knowledge available to the NN.

The pre-trained CNN used in this research was ResNet-18 [35], which is pre-trained on the ImageNet database [36]. At only 18 layers deep, it is relatively lean, with training and subsequently running the CNN thereby requiring less time and processing power than its larger counterparts. TL was used to retrain the CNN on the Google Colab cloud platform using Python and the Fastai library [37]. Since a Google Colab Pro membership was used, the graphical processor unit used in this work was either a NVIDIA V100 or A100, depending on availability. All CNNs described herein were trained using a batch size of 16 and for 14 epochs using the fit.one_cycle function. This method of training is an implementation of cyclical learning rate [38,39] and super-convergence [40] principles. Practically, this means that the hyperparameters of learning rate, momentum and weight decay are automatically determined, leading to CNNs that can outperform those created using traditional hyperparameter tuning techniques for some applications [40]. In this work, it is sufficient to use accuracy as the performance metric, as the data is *balanced*, meaning each class contains the same number of responses [41]. During the analysis of the data, in some instances, the data may be split in such a way that some classes contain one more or less response than the others. For example, the 10% test set split of all 19,872 responses results in 1987.2, as such the resulting test set contains 1987 responses. This is a common occurrence and will introduce a negligible bias into the accuracy-based performance assessment, whereas for larger class imbalances roughly ranging up from a two times difference, other performance metrics become more reliable, such as F1-score [42].

## 4. Response Classification Performance Assessment

This section will assess the performance of this vibro-acoustic object recognition technique in two main regards: firstly, the ability to distinguish all 23 objects from one another and secondly, the ability of this technique to generalise across broader object classes to recognise never-before-seen instances of an object class.

### 4.1. The Effects of Pre-Processing and Training Set Size on Classification Accuracy

This subsection will focus on the effects of the data pre-processing and training set size on the classification accuracy. Here, 864 responses for each of the 23 objects in Figure 1 were used to generate the four data types shown in Figure 4. This yields 19,872 images for each of the four pre-processing techniques, labelled according to Figure 1 using numbered file names. Of these, 10% are kept aside as a test set to be used later to determine the accuracy of each CNN. This test set is comprised of the same 1988 responses for each technique, but with different pre-processing applied. Since 1988 is not a multiple of 23, there are either 86 or 87 responses for each object class. The remaining 17,884 images are used as the training set. To simulate smaller data sets, the number of responses is also reduced by the factor m=1,2,4,6,8,10. Over the six training set sizes and four pre-processing techniques, there are a total of 24 data sets used for this section.

**Figure 6:**
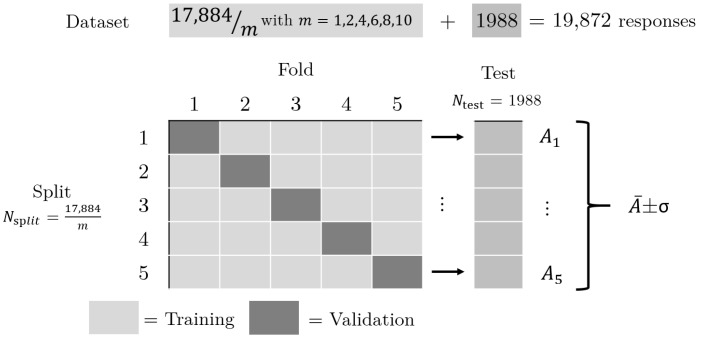
A depiction of the five-fold cross-validation procedure used to compare the four pre-processing techniques at six sample sizes. *m* represents the fraction of the data set used in the five-fold cross-validation, *A* is the accuracy obtained for each split, $\bar{A}$ is the mean accuracy of all five splits, σ is the associated standard deviation, $N_{split}$ is the total size of that split of the training data set and $N_{test}$ is the test set size.

A 10% randomly selected stratified sample was set aside as the test set for each pre-processing type to subsequently evaluate the performance of the trained CNNs. Five-fold cross-validation was then applied to rigorously compare the effects of the four pre-processing techniques on the CNN performance. This divides the remaining data into five “folds”, four of which are used to train the CNN, with the remaining fold used to validate its performance. This process is repeated five times to generate five separate CNNs for each data set, each of which uses a different fold as the validation set. This means that every piece of data not in the test set is used to both train and validate the CNNs, generating a total of 120 CNNs for the 24 data sets. For a fair comparison, all CNNs for all values of *m* must be compared against the same test set for each pre-processing type. Therefore, the metrics presented in this section were always generated using the same test set, but with differing pre-processing applied for the four techniques. This procedure is depicted in Figure 6.

**Figure 7:**
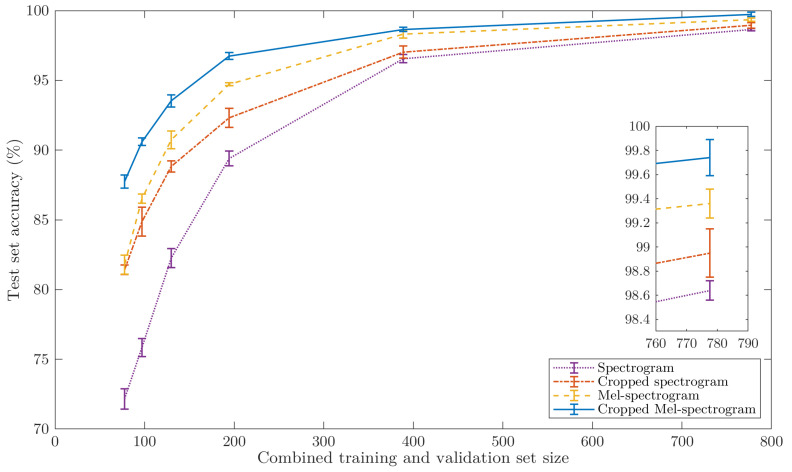
The accuracy and the associated standard deviations of the CNNs utilising the four data types as the combined size of the training and validation set is decreased. An expanded plot representing four data points is overlaid to the right.

The results of the five-fold cross-validation for the 24 data sets can be seen in Figure 7. The overall trend for all four pre-processing techniques is that a larger sample size yields a higher accuracy as all four techniques tend towards 100%. However, as the sample size decreases, the performances of four techniques begin to diverge. The overall worst performing CNNs used spectrograms, reaching as low as 72.16% for 78 responses used per object. The second-best technique was the cropped spectrogram; however, it performed fairly similarly to mel-spectrograms, with a maximum difference in performance of only about 2%. Finally, the CNNs utilising cropped-mel-spectrograms performed universally best of the four techniques and exhibited a considerably lower sensitivity to sample size than the other techniques, while also being the most accurate when the entire data set was used. The mel-spectrograms obtained an accuracy of 87.74% while only being trained on 78 responses per object. This training set size is arguably small enough to make manual data acquisition a viable alternative to the bespoke automated arrangement used here.

The expanded plot in Figure 7 shows more clearly the results at the largest sample size. It can be seen that all four techniques obtained a performance above 98.64% with non-overlapping standard deviations. Overall, spectrogram CNNs performed the worst; however, the cropped spectrograms CNNs gained a performance boost of over one percentage point. Similarly, cropped mel-spectrogram CNNs outperformed mel-spectrogram CNNs by about half a percent. Overall, cropping seems to have the expected effect on the performance, as the CNNs can generalise more effectively if there is less irrelevant information within each image. Similarly, mel-spectrograms perform better than spectrograms, likely caused by the higher density of object resonances at the lower frequencies, where mel-spectrograms devote more image area, therefore allowing the CNNs to generalise more effectively. Thus, the remainder of this paper focuses on cropped mel-spectrograms as the primary datatype with the full data set for the CNNs, as their classification accuracy exceeds that of the others.

**Figure 8:**
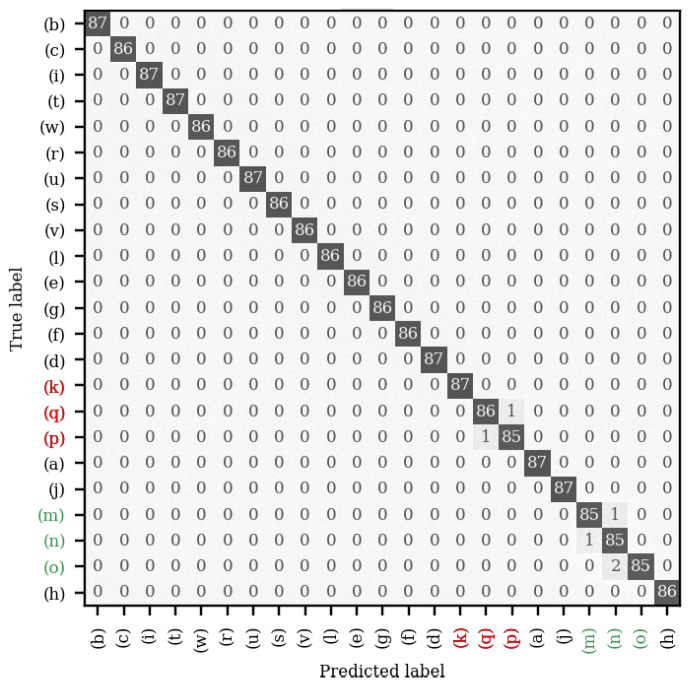
The confusion matrix for one of the five mel-spectrogram CNNs making predictions on the test set. Highlighted for convenience are the two groups of objects where erroneous predictions were made: in red, (k), (p) and (q) are the table tennis balls; in green, (m), (n) and (o) are the tennis balls.

Figure 8 shows the confusion matrix for one of the five mel-spectrogram CNNs using the full data set. A confusion matrix with 100% accuracy would only have predictions lying on the diagonal from the top left to the bottom right, as this corresponds to the predicted label being equal to the true label. From this, it can be seen that the CNN made errors in two main regions of the confusion matrix, that is, two errors where it mistook the table tennis balls (p) and (q) for one another once and when it mistook the tennis balls (m), (n), and (o) for one another a further four times. These errors could likely be removed by including a larger data set, allowing the CNN to generalise more effectively; by using a larger CNN, such as ResNet-34, enabling the extraction of more complex features; or by increasing the spectral range and spectral resolution of the spectrograms, allowing the identification of more features. Despite the potential improvements, in its current form, the performance of this CNN rivals existing *contact* object recognition techniques. Direct comparison to existing work is difficult, as they all utilise a different number of objects (ranging from 12 to 50) with some using filled containers [21,23] rather than solid objects [22], or both [20]. However, these can be summarised as performing from within the range of 85.5% [23] up to 98.2% [22], with the highest performing work using the most similar objects to those used in this work. This contextualises the technique presented herein as a viable alternative, not only increasing recognition accuracy, but also introducing non-contact functionality.

### 4.2. Sensitivity to Broader Object Classes

While the previous subsection has shown that it is possible to recognise specific objects using their vibrational response to an acoustic excitation, it has not yet been established if this technique can be used to recognise a broader class of similar objects, for example, to recognise any soda can rather than a specific soda can. The few errors shown in Figure 8 are a subtle indicator that, acoustically, these four groups of like objects may share some features; hence why a person can easily distinguish the sound of any table tennis ball bounce to that of any other type of ball. As such, the ability of the system to classify objects into broader classes is the focus of this section. To do this, the CNN must be able to generalise features related to all the included soda cans and be able to correctly identify features of an unseen soda can. As such, this section utilises the grouped objects: table tennis balls—objects (k), (p) and (q); tennis balls—objects (m), (n) and (o); empty soda cans—objects (r), (s), (t); and the sealed soda cans—objects (u), (v), (w). The CNN was trained on two of the three objects within each group, labelled as the same class, as well as all of the previous objects, minimising the likelihood that the results occur due to chance. This means that the accuracy can then be obtained using the third (unseen) object within each group. This experiment will show that the CNN is able to generalise features common to all three objects within each group, despite having seen only two of them.

**Figure 9:**
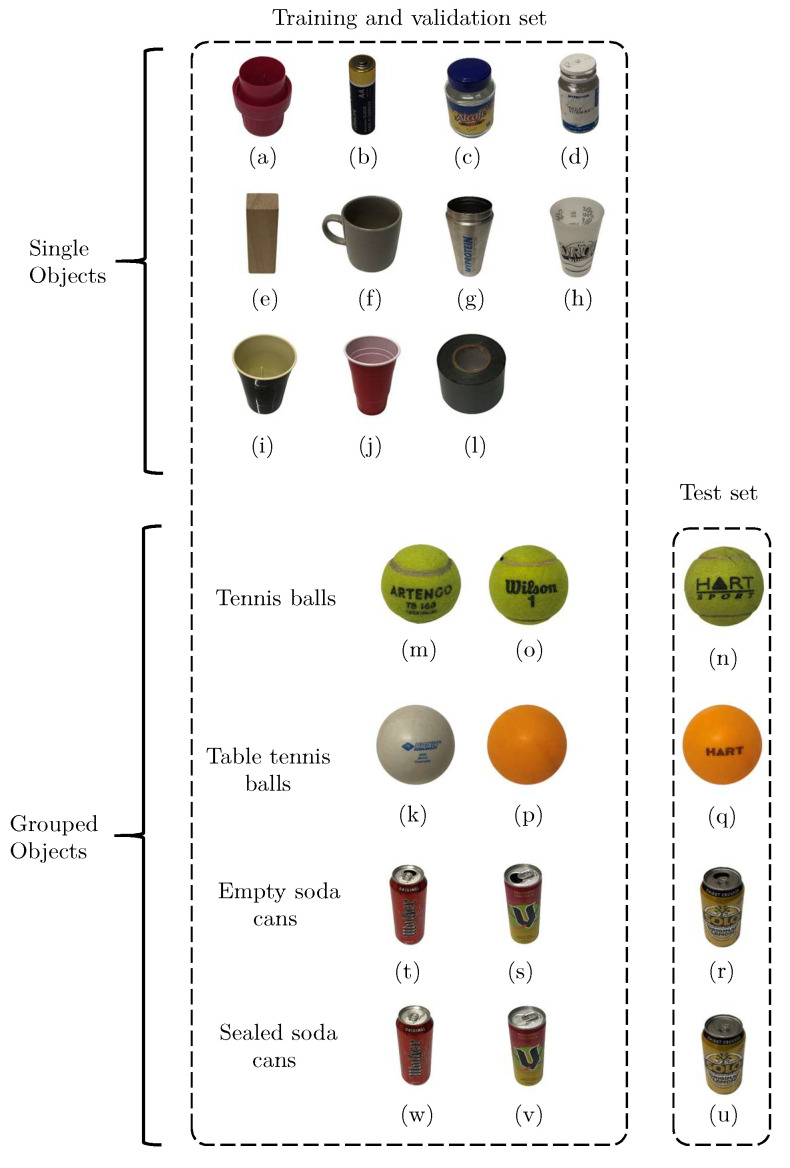
The 23 objects labelled as single objects or grouped objects. Here, there are four groups of objects, tennis balls, table tennis balls and either full or empty soda cans. Each object in this represents 864 mel-spectrograms.

Figure 9 illustrates how the data were labelled for this section, achieved by modifying the file names, through which the classes are assigned. Here, a new ResNet-18 CNN was trained using 864 cropped mel-spectrograms for each of the single objects, as per the previous section, but also on four pairs of objects belonging to each of the four groups, again with 864 scans per object. The box in the figure titled “training and validation set” was split into the standard portions of 80% training set and 20% validation set. However, since the performance metric of interest here is how accurately the CNN can classify never-before-seen items belonging to a specific group, the test set consists only of the four hold-out objects.

**Figure 10:**
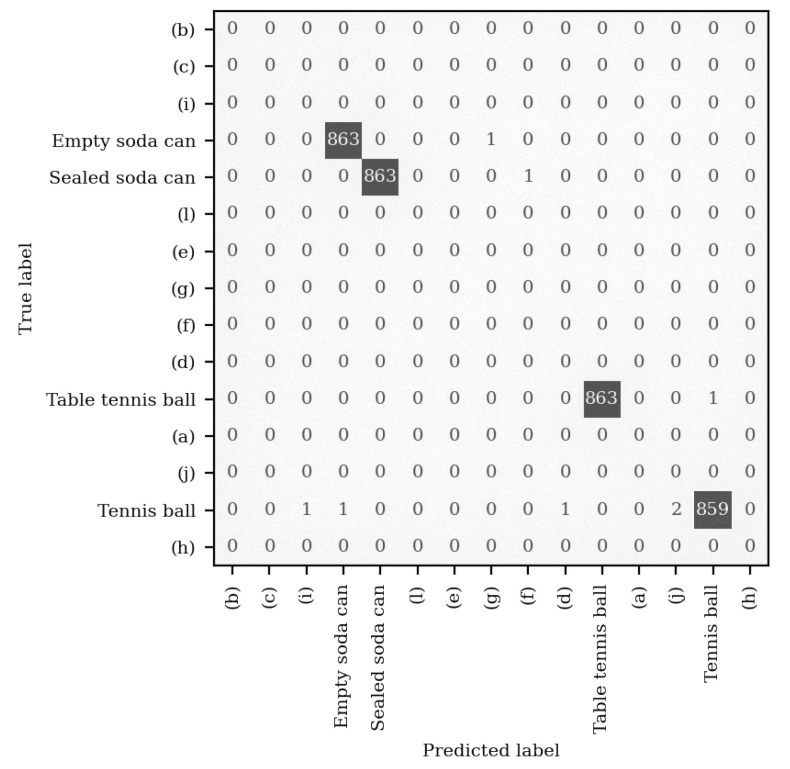
The confusion matrix of the test set used to assess the ability of the technique to detect broader classes of objects.

The confusion matrix for this test set can be seen in Figure 10. Overall, the CNN correctly predicted the class of the hold-out object 99.83% of the time. Unsurprisingly, the most common prediction error was between the full and empty cans, but this is still a comparatively small number of mistakes when compared to the number of correct predictions and would likely decrease if the CNN was given more than two soda cans during the training phase. There is one cited work which similarly looks at broader object classes rather than classifying individual objects [20]. In that work, only three object classes were excited using a simple shake actuator, consisting of filled bottles of water, pieces of paper and, finally, rigid objects, which make no noise. This work showed that a never-before-seen object belonging to the broader object classes could be recognised with an accuracy of up to 95.8%. However, with such a small number of classes in the data, there is a 33.3% chance that the classifier can obtain the correct output. Similarly, the chosen objects do not represent the most challenging selection. Regardless, the non-contact acoustic object recognition technique presented herein outperforms this technique, obtaining near-perfect classification despite a total of 15 potential class outputs.

## 5. Conclusions and Future Work

This work aimed to show that the applications of non-contact vibro-acoustic measurement using a loudspeaker and a laser Doppler vibrometer (LDV) could be expanded to object recognition. This technique can rapidly recognise previously characterised objects within seconds by exciting a small vibrational response using a loudspeaker measured using an LDV and classified using a convolutional neural network (CNN), which was trained using transfer learning based on ResNet-18. The technique was developed and verified using 23 household objects; however, it is not only limited to household objects. A bespoke, automated vibro-acoustic response measurement system was developed specifically to enable the rapid collection of quality raw time data. These were then pre-processed into images for use with the CNN as four data types: spectrograms and mel-spectrograms, with and without cropping the image above the excitation frequency.

The paper first looked at how the pre-processing approach and training set size may affect the accuracy of the object classification predictions. From this, three main observations can be made. Firstly, all the CNNs’ accuracies increased for larger training set sizes; this is not surprising, as it is a fairly typical outcome. Secondly, the CNNs utilising mel-spectrogram inputs performed better than those utilising spectrograms. This is likely due to the higher density of resonances at the lower frequencies, therefore dedicating more space to these in the spectrogram image and allowing for more effective classification and generalisation. Lastly, removing any spectral information in the scan above the frequency being excited at that time instant (cropping) increased the accuracy. This is likely due to the cropped region containing mostly noise, which inhibits the CNN’s ability to effectively generalise. While all the pre-processing techniques lead to a sufficiently high accuracy when the training set size was at its largest, the use of cropped mel-spectrograms outperformed the others with an accuracy of 99.74 ± 0.15% on the test set. As such, only the full mel-spectrogram data set was used to train CNNs in the subsequent section.

While the aforementioned performance is near-perfect, a practically viable object recognition technique must be able to detect broader classes of objects, not just specific ones, for example, all soda cans, not just one specific soda can. To test this, four triplets of similar objects were grouped together so that the CNN could generalise across them. When the CNNs predicted the class of the never-before-seen third item in the groups, an accuracy of 99.83% was obtained, confirming that this approach constitutes a practically viable object detection technique. It is important to note that when generalising to unseen objects, the broader object class must contain some vibro-acoustic similarities; however, it can be reasonably expected that a sufficiently large and diverse training set should still yield performance comparable to that described here.

While this paper has focused on object recognition, it has been shown that this technique is highly sensitive to slight differences in the objects, while also being capable of learning broader object classes. These characteristics open up many potential applications within various other fields. For example, this technique could be used for defect detection in production lines, where physical changes in an item will subtly modify its acoustic fingerprint. This is useful when all that is of concern is that a defect is present in an item, not what exactly the defect is; similar to the role checksums play in computing. Therefore, rather than manually inspecting parts for defects, the objects may be excited to ensure the vibrational response is within the limits of “good” responses contained in the training data. Similarly, the response of objects containing a defect may constitute another class, such that they may be similarly detected and discarded. This application may even be simpler than the object detection tasks described here if the parts on the production line all share the same orientation and locations on the production line, therefore allowing both the training data and the classification measurement to be conducted from the same point on the object and removing the need for the rotary stage during the acquisition of training data.

Another potential application is the identification of loose rocks in underground mines. Current techniques depend on a worker tapping the roof with a bar and listening to the sound to determine if it is loose, known as “roof sounding” [43]. A more high-tech solution employs the use of a vibration sensor, which is similarly tapped on the roof to identify loose rock [44]. However, both require workers enter the newly created area. Therefore, the introduction of a non-contact remote alternative technique would reduce the risk to human life. Here, the roof can be excited remotely using either acoustic or seismic techniques (for example, tapping the walls from a safe distance), while the SLDV remotely measures the response of various locations on the roof. Training data would be required to be labelled as either “secure” or “loose”. The collection of such data may be the most challenging aspect, potentially requiring a skilled roof sounder to assist. With the performance described herein, this technique could likely match that of a skilled roof sounder, potentially outperforming them if higher-quality training data could be acquired by alternative means.

Despite the technique’s promising performance, there remain some aspects of the system that require further investigation and refinement. Firstly, the loud audible chirp used for exciting the response in the samples makes the system unpleasant to nearby people. Therefore, it is important to use non-audible frequencies to excite the target. Secondly, to increase the range, the energy of the speaker should be directed in the approximate direction of the target, rather than being lost to the surroundings. Therefore, integrating an ultrasonic parametric speaker to perform the excitation is an ideal next step for the system, with an investigation into other excitation signals and frequency ranges. Then, an in-depth investigation into the effects of object distance and sound reflections can be conducted. Finally, the automatic acquisition procedure could be modified to accommodate larger objects with more complex geometries that may not fit onto a rotary stage.

## Data Availability

The 19,872 object responses can be found online at Mendeley data: https://data.mendeley.com/datasets/7km4xcx5hb/2, accessed on 21 November 2022.

## Abbreviations

The following abbreviations are used in this manuscript:

LDV	Laser Doppler vibrometer
SLDV	Scanning laser Doppler vibrometer
AV	Autonomous vehicle
NN	Neural network
CNN	Convolutional neural network
TL	Transfer learning
